# Comparing denominator degrees of freedom approximations for the generalized linear mixed model in analyzing binary outcome in small sample cluster-randomized trials

**DOI:** 10.1186/s12874-015-0026-x

**Published:** 2015-04-23

**Authors:** Peng Li, David T Redden

**Affiliations:** Department of Biostatistics, School of Public Health, University of Alabama at Birmingham, Birmingham, Alabama 35294 USA

**Keywords:** Wald *F* test, Type I error, Power

## Abstract

**Background:**

Small number of clusters and large variation of cluster sizes commonly exist in cluster-randomized trials (CRTs) and are often the critical factors affecting the validity and efficiency of statistical analyses. *F* tests are commonly used in the generalized linear mixed model (GLMM) to test intervention effects in CRTs. The most challenging issue for the approximate Wald *F* test is the estimation of the denominator degrees of freedom (DDF). Some DDF approximation methods have been proposed, but their small sample performances in analysing binary outcomes in CRTs with few heterogeneous clusters are not well studied.

**Methods:**

The small sample performances of five DDF approximations for the *F* test are compared and contrasted under CRT frameworks with simulations. Specifically, we illustrate how the intraclass correlation (ICC), sample size, and the variation of cluster sizes affect the type I error and statistical power when different DDF approximation methods in GLMM are used to test intervention effect in CRTs with binary outcomes. The results are also illustrated using a real CRT dataset.

**Results:**

Our simulation results suggest that the Between-Within method maintains the nominal type I error rates even when the total number of clusters is as low as 10 and is robust to the variation of the cluster sizes. The Residual and Containment methods have inflated type I error rates when the cluster number is small (<30) and the inflation becomes more severe with increased variation in cluster sizes. In contrast, the Satterthwaite and Kenward-Roger methods can provide tests with very conservative type I error rates when the total cluster number is small (<30) and the conservativeness becomes more severe as variation in cluster sizes increases. Our simulations also suggest that the Between-Within method is statistically more powerful than the Satterthwaite or Kenward-Roger method in analysing CRTs with heterogeneous cluster sizes, especially when the cluster number is small.

**Conclusion:**

We conclude that the Between-Within denominator degrees of freedom approximation method for *F* tests should be recommended when the GLMM is used in analysing CRTs with binary outcomes and few heterogeneous clusters, due to its type I error properties and relatively higher power.

## Background

Cluster-randomized trials (CRTs), also called group-randomized trials, are widely used in the evaluation of interventions in health services research [1]. CRTs are distinct from other randomized controlled trials in that the identifiable clusters of subjects/participants such as medical practices, hospital wards, schools, or communities, rather than individuals, are randomly assigned to different intervention conditions [2]. Because the clusters are formed not at random but rather through some connections among their members, a positive intraclass correlation (ICC, denoted as *ρ*) [3] among observations in the same cluster is expected. Although typically the ICC is small (*ρ* < 0.05) [4] and not known when a trial is planned, the adjustment for ICC is necessary for a valid statistical analysis at the subject level. Any statistical test ignoring the non-independence of participants within clusters will underestimate the variances of the intervention effects and consequently inflate the type I error rates [5]. CRTs can be analyzed at the cluster level, by deriving summary statistics for each cluster, or at the individual level using the data for each participant in each cluster [1]; however, only the individual-level analyses enable the adjustment of the participant characteristics to minimize the selection bias. Two modeling approaches are commonly used for the individual-level analyses of CRTs with the consideration of clustering. One is the random effects model or generalized linear mixed model (GLMM), which incorporates random effects to reflect the correlation among observations of same cluster [6]; the other is the marginal or population mean model using the generalized estimating equations (GEE) approach [7]. These two modeling methods should provide similar results if both models are correctly specified and their underlying assumptions hold well, while the interpretation of the fixed effects estimates is a little different [8]. The GLMM is more complicated and informative than the GEE approach by providing the estimation of the variance components, which are otherwise treated as nuisance parameters in GEE [7]. The choice of modeling method should depend on the scientific questions and the validity of the underlying assumptions. In cases where heterogeneity is of significant interest, the GLMM could be the better choice. In addition, the pattern of missing data, which is common in most trials, is another important consideration on the model selection. The GLMM is valid under both missing completely at random (MCAR) and missing at random (MAR), while the GEE approach is valid only under MCAR even though some imputation strategies have been proposed for valid GEE inference under MAR [8].

The GLMM combines the properties of two statistical models that are widely used in different fields: generalized linear models (GLMs) which handle non-normal data from the exponential family by using link functions and linear mixed models (LMMs) which incorporate random effects [8]. In the GLMM, the Wald statistics are recommended to test the null hypothesis of fixed effects because the likelihood ratio tests are unreliable for small to moderate sample sizes [8-10]. Wald statistics are calculated by dividing parameter estimates or linear combinations of parameter estimates by their estimated standard errors. In the GLMM, the approximated Wald *F* test, rather than Chi-squared test, is recommended to handle finite sample sizes and overdispersion, which commonly occurs for binary or Poisson regression models, since the variance of both distributions is a function of the mean [8]. The most challenging issue for the approximated Wald *F* test is the estimation of the denominator degrees of freedom (DDF). It is expected that overestimation of DDF will produce a liberal test leading to inflated type I error and the underestimation of DDF will produce a conservative test leading to the potential power loss. In practice, five DDF approximations are used, including Residual DDF, Containment DDF, Between-Within (B-W) DDF, Satterthwaite DDF and Kenward-Roger (K-R) DDF for the Wald *F* test; however, none of them work well in all situations and some are only valid in very strict conditions [8,9,11]. Simulation studies [12-15] under unbalanced split-plot designs have shown that the K-R DDF approximation has the best performance in preserving the nominal type I error; and that the covariance structure, the sample size, and the degree of imbalance are the major factors that affect the performance. Although K-R DDF approximation is recommended to maintain the type I error rate, its small sample performance was evaluated mainly on normal-distributed outcomes under repeated measures designs [14,15]. CRTs typically have characteristics including small cluster numbers, moderate to large variable cluster sizes, and weakly correlated outcomes within the same cluster (*ρ* < 0.05) [4]. These characteristics are quite different from those encountered in repeated measure designs. Therefore, the validity of the K-R DDF approximation for non-normal outcomes under CRT scenarios needs further evaluation.

The purpose of the present study is to compare and contrast the statistical properties of the five DDF approximation methods for GLMM when testing intervention effects for binary outcomes in CRTs with a small number of clusters. Specifically, the type I error rates to test the null hypothesis of treatment effect are examined for each of five DDF approximation methods (Containment, Residual, B-W, Satterthwaite, and K-R) under situations with different ICCs, sample sizes, and cluster size variation. For the methods that can maintain the nominal type I error rate, statistical power is compared. Because the compound symmetry is the reasonable and most widely accepted variance-covariance structure for CRT data, it is the only variance-covariance structure considered in this study.

## Methods

### Generalized linear mixed models and Wald *F* test

GLMM is the extension of GLM by introducing random effects into the linear predictor of the GLM [16,17]. Let *K*, *n*_*i*_ denote the number of clusters and the number of observations in cluster *i*, respectively, the model with *p* predictors can be expressed as:

1 $$ {Y}_i={g}^{-1}\left({X}_i\beta +{Z}_i{b}_i\right)+{\epsilon}_i\kern0.75em i=1,\dots, K $$

where

*Y*_*i*_ is the *n*_*i*_ × 1 response vector for the *i*^*th*^ cluster;

*g*^− 1^(·) is the inverse of a differentiable monotonic link function;

*X*_*i*_ is a *n*_*i*_ × *p* matrix of fixed covariates;

*β* is a *p* × 1 vector of fixed-effects regression parameters;

*Z*_*i*_ is a *n*_*i*_ × *v* design matrix of random effects, where *v* is a design parameter;

*b*_*i*_ is a *v* × 1 vector of cluster-specific random effects;

*ϵ*_*i*_ is a *n*_*i*_ × 1 error vector.

The parameters in GLMM can be estimated either by the standard maximum likelihood (ML) estimation, which estimates the standard deviations of the random effects assuming that the fixed effect estimates are precisely correct, or by the restricted maximum likelihood (REML) estimation, a variant that averages over some of the uncertainty in the fixed-effect parameters [8,11].

The Wald test is commonly used for hypothesis testing in GLMMs. To test the fixed effects *H*_*o*_ : *Lβ* = 0, the Wald large sample Chi-squared test is given as

2 $$ {T}^2\left(\widehat{\beta}\right)={\widehat{\beta}}^{\prime }{L}^{\prime }{\left(L\widehat{Y}\left(\widehat{\beta}\right){L}^{\prime}\right)}^{-1}L\widehat{\beta} $$

where *L* is *r* × *p* matrix with rank *r* ≤ *p* for the general linear hypothesis, $\widehat{\beta}$ is the estimate of *β* by some estimation technique and $\widehat{Y}\left(\widehat{\beta}\right)$ is the estimated variance-covariance matrix of $\widehat{\beta}$. However, a more conservative Wald *F* test is preferred in GLMMs to handle finite samples and overdispersion:

3 $$ F\left(\widehat{\beta}\right)={T}^2\left(\widehat{\beta}\right)/r $$

with *r* numerator degrees of freedom and an approximated DDF, say *d*. Suppose we are going to test the null hypothesis of no intervention effect, the Wald *F* statistic $F\left({\widehat{\beta}}_T\right)$ will have an approximated *F* distribution with 1 numerator degrees of freedom and *d* DDF which must be specified or estimated. Five DDF approximations are proposed to justify the correlated outcomes and briefly discussed below.

#### Residual DDF

The simplest method for the DDF estimation is the Residual method which is calculated by *N* − *rank*[*X*], where N=${\displaystyle {\sum}_{i=1}^K\;}{n}_i$, the total participants across all clusters.

#### Containment DDF

The Containment method chooses DDF as the smallest rank contribution of the random effects that contain the fixed effects to the design matrix in split-plot design [15]. This choice of DDF matches the tests performed for balanced designs and could be adequate for moderately unbalanced designs [15]. Under the framework of CRTs, if the treatment effect is fixed and not contained in any random effects, the Containment DDF is calculated by *N* − *K*.

#### Between-Within DDF

Schluchter and Elashoff [18] divide the residual degrees of freedom into between-cluster and within-cluster portions and suggest that in a mixed model, if a fixed effect changes within any cluster, within-cluster degrees of freedom should be assigned to the effect; otherwise, the between-cluster degrees of freedom should be assigned to the effect. In a CRT to test the intervention effect across the clusters, the between-cluster degrees of freedom will be applied and calculated as *K* − *rank*[*X*].

#### Satterthwaite DDF

Fai and Cornelius [13], follow Satterthwaite’ premise [19] to propose a method for multi-degree-of-freedom tests in unbalanced split-plot design. The degrees of freedom are calculated as a function of the variance of the parameter estimate. Briefly, ${\left(L\widehat{Y}\left(\widehat{\beta}\right){L}^{\prime}\right)}^{-1}$ is decomposed to yield ${P}^{\prime }{\left(L\widehat{Y}\left(\widehat{\beta}\right){L}^{\prime}\right)}^{-1}P= diag\left({\lambda}_m\ \right)$ where columns of *P* are normalized eigenvectors and the *λ*_*m*_ are the corresponding eigenvalues of ${\left(L\widehat{Y}\left(\widehat{\beta}\right){L}^{\prime}\right)}^{-1}$. Let *Q* = *rF*, using the decomposition, $Q={\displaystyle {\sum}_{m=1}^r}\frac{{\left({p}_m^{\prime }L\widehat{\beta}\right)}^2}{\lambda_m}={\displaystyle {\sum}_{m=1}^r}\kern0.22em {t}_{U_m}^2$, the sum of *r* approximate *t* variables squared, where ${p}_m^{\prime }$ is the *m*^*th*^ eigenvector and *U*_*m*_ is the approximate degrees of freedom for the *m*^*th*^ independent single degree of freedom *t* statistic. Since $\frac{Q}{r}\sim {F}_{r,d}$, *d* can be solved using the relationship $E(F)=\frac{d}{d-2}$. For *r* > 1, $d=\frac{2E\left[Q\right]}{E\left[Q\right]-r}$, and for *r* = 1, $d=\frac{2{\left(L\widehat{Y}\left(\widehat{\beta}\right){L}^{\prime}\right)}^2}{Var\left[L\widehat{Y}\left(\widehat{\beta}\right){L}^{\prime}\right]}$, where $Var\left[L\widehat{Y}\left(\widehat{\beta}\right){L}^{\prime}\right]$ is approximated using the multivariate delta method.

#### Kenward-Roger DDF

Kenward and Roger [14] propose a scaled Wald statistic ${F}^{*}={T}^2\left(\widehat{\beta}\right)/\varphi r$ for mixed model for small samples. An appropriate *F*_*r,d*_ approximation to the sampling distribution of F* is derived with the Satterthwaite method by matching the first two moments of F* with those from the approximating *F* distribution and solving for the values of *φ* and *d*:

4 $$ E\left[{F}^{*}\right]=E\left[{F}_{r,d}\right]=\frac{d}{d-2}. $$

5 $$ Var\left[{F}^{*}\right]=Var\left[{F}_{r,d}\right]=2{\left(\frac{d}{d-2}\right)}^2\frac{r+d-2}{r\left(d-4\right)}. $$

The value of *d* thus derived is the K-R DDF. For *r* =1, K-R DDF is the same as Satterthwaite DDF, but the K-R approximation generates a more conservative test by inflating the variance-covariance matrix, by *φ*.

### Data simulation

We conducted simulation studies based on a two-armed CRT design with binary outcomes. For simplicity but without the loss of generalizability, we assume the control and the intervention arms contain equal number of clusters and no covariates. Correlated binary responses are generated using a Beta-binomial method [20], by which the proportion of events in a cluster is a random draw from Beta(a,b). It can be shown that the marginal proportion of events in a cluster is defined as $\mu =\frac{a}{a+b}$, and the ICC is $\rho =\frac{1}{1+a+b}$. A logistic regression model is used for the marginal mean of *y*_*ij*_

6 $$ logit\left({\mu}_{ij}\Big|{x}_i\right)=\alpha +\beta *{x}_i+{b}_i, \kern3em {b}_i\sim N\left(0,\;{\tau}^2\right), $$

where *x*_*i*_ is a cluster-level binary predictor indicating the treatment arms (*x*_*i*_ = 0 for control and *x*_*i*_ = 1 for active intervention), *i* = 1, …, *K* and *j* = 1, …, *n*_*i*_ and the *b*_*i*_ are assumed to be normally distributed. The marginal mean was set as {*μ*_*i*_|*x*_*i*_ = 0} = 0.25. The *τ* is determined by the Beta-binomial method with defined marginal mean and selected intraclass correlation (*ρ*)*.* Under our simulation settings, the approximated *τ*^2^ are 0.12, 0.17, 0.44 and 0.81 for ICCs equal to 0.001, 0.01, 0.05 and 0.1, respectively. These ICC values reflect levels often seen in practice [4,5,21]. To examine whether the Beta-binomial method was generating normally distributed *b*_*i*_ on the logit scale, Q-Q plots were generated for the data simulations. The Q-Q plots of the *b*_*i*_ suggest that the normal distribution assumption holds. The sample sizes in our simulations are set as 10, 20, and 30 total clusters (*K*) with 20, 50, and 100 observations on average per cluster $\left(\overline{n}\right)$. The exact number of observations, *n*_*i*_, for each cluster *i* = 1, …, *K*, is randomly drawn and rounded from normal distributions with the mean equal to $\overline{n}$ and variance equal to *σ*^2^. The variation of cluster sizes can be measured by the coefficient of variation (*cv*), which is the ratio of standard deviation of the cluster sizes over the mean of the cluster sizes. So, we set ${\sigma}^2={\overline{n}}^2c{v}^2$. In our simulations, *cv* is at the range of 0 to 1. To avoid the impossible situation that the number of observations in a cluster is negative or zero, we bound the smallest cluster size to 1. Under these settings, the DDF by different approximation methods are listed in Table 1. For each scenario, 5000 independent replicates are generated for the type I error calculation, and 1000 independent replicates for power calculation. All simulations and analyses are conducted using SAS 9.3 (Cary, NC).

**Table 1:** **The denominator degrees of freedom of GLMM** **Wald** *F* **test by different approximation methods in the simulations under the framework of CRTs**

**Methods**	**Estimated denominator degrees of freedom**
Residual	${\displaystyle \sum_{i=1}^K}{n}_i-2$
Containment	${\displaystyle \sum_{i=1}^K}{n}_i-K$
Between-Within	*K* − 2
Satterthwaite	*d*, estimated from data
Kenward-Roger	*d*, estimated from data

The type I error rate of each DDF approximation is calculated by computing the observed fraction of Wald *F* tests rejecting the null hypothesis (*H*_*o*_ : *β* = 0) when the null hypothesis is true. At the nominal 0.05 level and 5000 simulations, we expect the simulated type I error rate to be between 0.044 and 0.056 (95% confidence interval), and any procedure with type I error rate below this range will be considered conservative, above this range will be considered liberal, and within this range will be considered as having the nominal type I error rate. The power is calculated by computing the observed fraction of Wald *F* tests rejecting the null hypothesis (*H*_*o*_ : *β* = 0) when the true value of *β* is log1.5 (i.e., odds ratio is 1.5).

### Real data illustration

All the five DDF approximation methods are illustrated using a real CRT, investigating whether intervention in general practices improved subsequent attendance at breast screening among women who did not respond to their initial invitation in the Newham borough of East London [22,23]. Among the participating practices, 12 were randomized to the intervention group and 14 to the control group. The reception staff of the general practices allocated in the intervention group entailed training of to contact non-attenders for breast screening. Control practices were given no training or advice. A total of 995 women in the intervention practices and 1069 in the control practices were included in the trial. The outcome of interest was the attendance at breast screening among women who did not respond to their initial invitation for routine breast screening. The intervention practices generally had higher rates of attendance in comparison to those in the control practices, although the attendance rate varied considerably between practices. It should be noted that a key feature of this trial is the small number of clusters (*K* = 26) with highly variable cluster sizes (*cv* ≈ 0.71).

## Results

### Type I error rates of Wald *F* tests with different DDF approximations

In this study, we compare the small sample performance of five DDF approximation methods in GLMM to test the null hypothesis of intervention effect under the framework of CRTs with binary outcomes. Specifically, we illustrate how the ICC, sample size, and the variation of the cluster size affect the type I error control of five DDF approximation methods.

**Figure 1:**
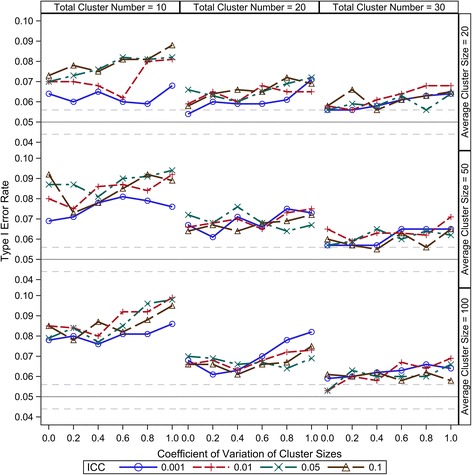
Observed type I error rates of GLMM Wald *F* test with Residual approximation of denominator degrees of freedom. The type I error rates are calculated from 5000 independent simulation replicates. The solid grey lines indicate the nominal level and the dashed grey lines indicate the upper and lower bounds of the 95% confident interval.

**Figure 2:**
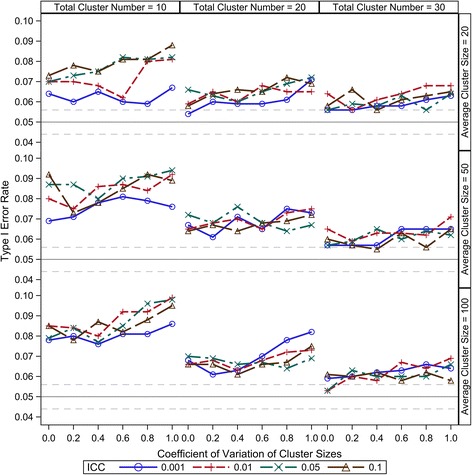
Observed type I error rates of GLMM Wald *F* test with Containment approximation of denominator degrees of freedom. The type I error rates are calculated from 5000 independent simulation replicates. The solid grey lines indicate the nominal level and the dashed grey lines indicate the upper and lower bounds of the 95% confident interval.

**Figure 3:**
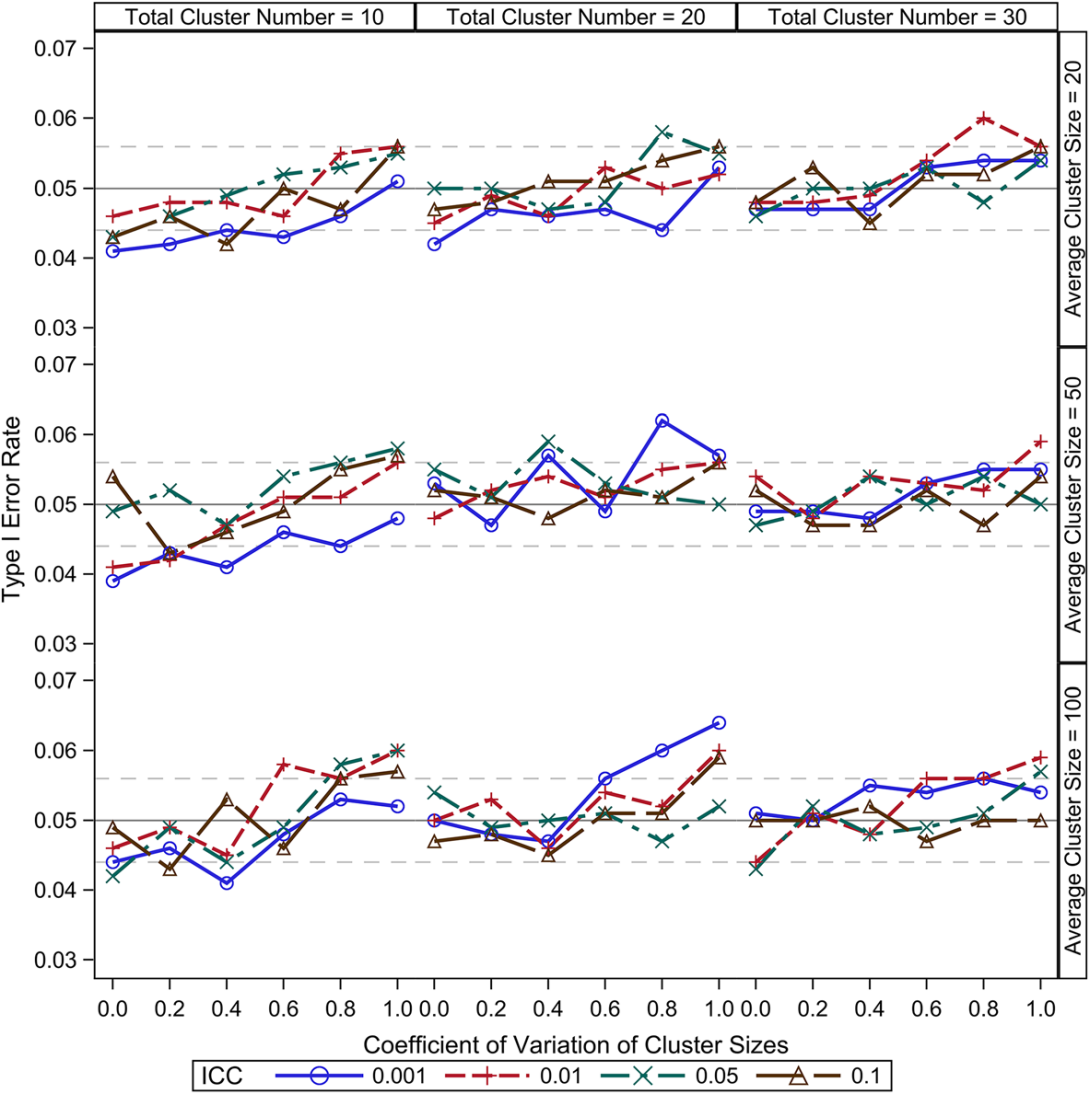
Observed type I error rates of GLMM Wald *F* test with Between-Within approximation of denominator degrees of freedom. The type I error rates are calculated from 5000 independent simulation replicates. The solid grey lines indicate the nominal level and the dashed grey lines indicate the upper and lower bounds of the 95% confident interval.

**Figure 4:**
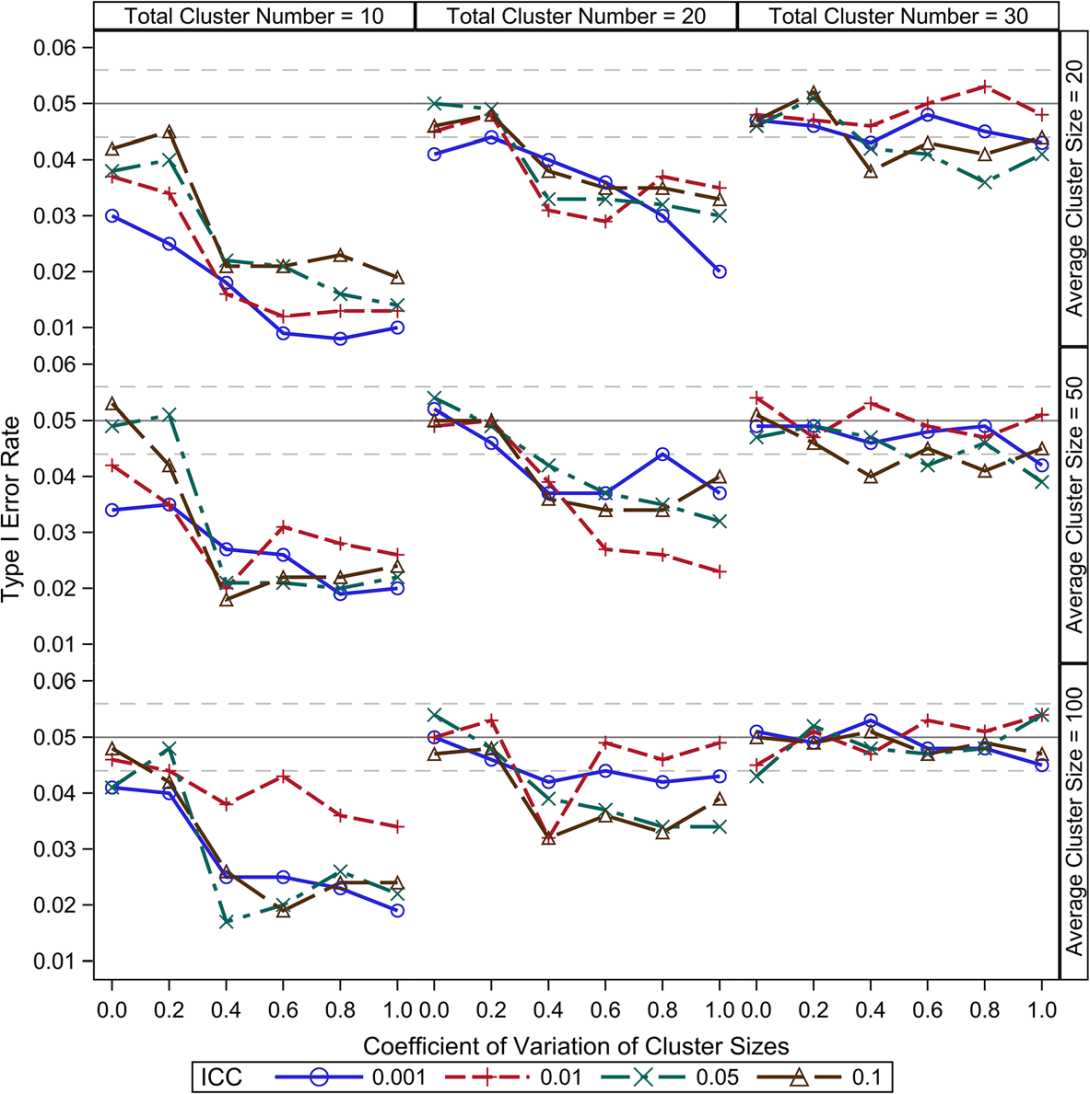
Observed type I error rates of GLMM Wald *F* test with Satterthwaite approximation of denominator degrees of freedom. The type I error rates are calculated from 5000 independent simulation replicates. The solid grey lines indicate the nominal level and the dashed grey lines indicate the upper and lower bounds of the 95% confident interval.

**Figure 5:**
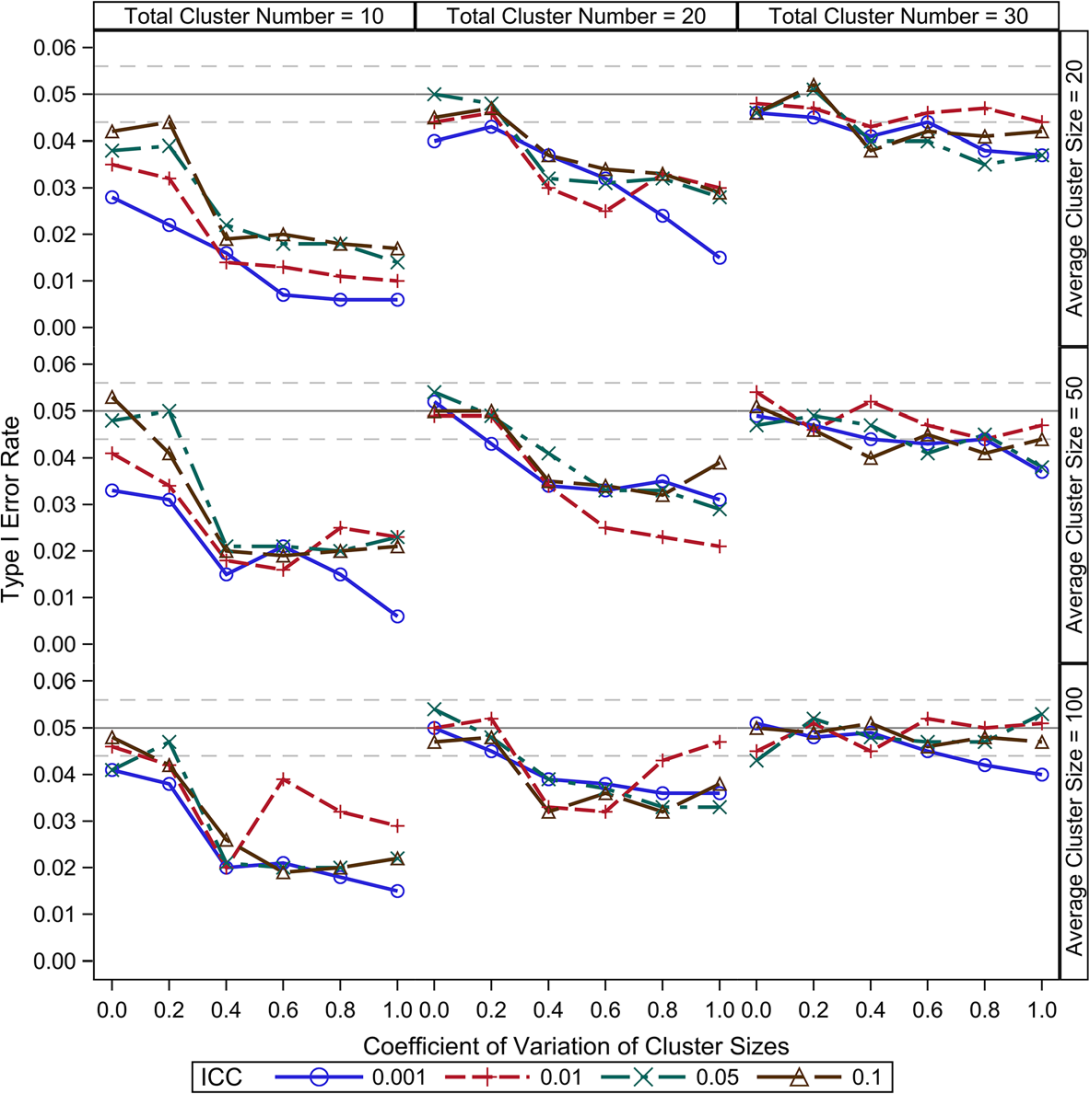
Observed type I error rates of GLMM Wald *F* test with Kenward-Roger approximation of denominator degrees of freedom. The type I error rates are calculated from 5000 independent simulation replicates. The solid grey lines indicate the nominal level and the dashed grey lines indicate the upper and lower bounds of the 95% confident interval.

Compound symmetry is a reasonable and most widely accepted variance-covariance structure for CRT data; therefore, it is the only consideration in this study. Under the range from 0.001 to 0.1, our results show that the ICC has little effect on the small sample type I error control of all the five DDF approximation methods (Figures 1, 2, 3, 4 and 5). There are two components of the sample size (*N*) in a CRT—number of clusters (*K*) and size of each cluster (*n*_*i*_) — and the relationship of $N={\displaystyle {\sum}_{i=1}^K}{n}_i$. As expected, larger *K* can keep the type I error rates closer to the nominal level for all the five DDF approximation methods (Figures 1, 2, 3, 4 and 5). Interestingly, the cluster size variation but not the average cluster size greatly affects the type I error rates of the DDF approximation methods in different ways (Figures 1, 2, 3, 4 and 5). However, the effects of cluster size variation can be diminished by increasing the total number of clusters. The variation of cluster sizes has little effect on the type I error rates when the number of clusters achieves 30.

The observed type I error rates of the Residual method to test the null hypotheses of intervention effect under various CRT scenarios are shown in Figure 1. The Residual DDF approximation does not consider the correlation of individuals among the same cluster and is calculated by subtracting 2 (the rank of X matrix in our settings) from the total number of individuals across all clusters. Clearly, the observed type I error rates of the Residual method are inflated when the total cluster number is less than 30. The inflation becomes more severe as the total cluster number becomes smaller and/or the variation of cluster size becomes larger. The inflation of type I error caused by the increased variation of cluster size can be diminished by increasing the cluster number; however, the Residual method cannot keep the observed type I error rate to the nominal level even for the equal cluster size (*cv* = 0); therefore, the Residual method should not be used in the GLMM analyses of CRTs if the cluster number is smaller than 30.

The observed type I error rates of the Containment method to test the null hypotheses of intervention effect under various CRT scenarios are shown in Figure 2. In the GLMM analyses of our CRT simulations, the intervention effect is set to be fixed and all the clusters have the common variance-covariance structure. Hence, the Containment method estimates the DDF as the total number of individuals across all clusters minus the number of clusters, i.e. *N* − *K*. Because of the large *N* and the relatively small *K*, the Containment method has the similar small sample performance to the Residual method regarding the inflated type I error rates. The observed type I error rates are inflated when the cluster number is smaller than 30; the inflation becomes more severe for smaller cluster numbers and/or for larger variations of cluster size. The inflation of type I error caused by the increased variation of cluster size can be diminished by increasing the cluster number, but not to nominal level given *K* < 30. Therefore, the Containment method should not be used in the GLMM analyses of CRTs with a cluster number smaller than 30.

The B-W method provides the optimal DDF approximation by providing the nominal type I error rate across our simulations, as shown in Figure 3. In most of the simulation situations, the observed type I error rates are located between 0.044 to 0.056, the 95% confidence interval of the nominal level, even when the number of clusters is as low as 10. Greater cluster size variation is associated with slight increases in the observed type I error rate when the number of clusters is small, such as *K* < 30. The Wald *F* test with B-W approximation tends to be slightly conservative under balanced design (*cv* = 0) and slightly liberal when the variation of cluster sizes is very high (*cv* > 0.8); however, the observed type I error rates under these extreme conditions are still very close to the nominal level.

The Satterthwaite method is intended as an accurate *F* test approximation and solves the DDF by matching the moments of observed Wald *F* statistics and an exact *F* distribution. Its type I error rate under various CRT scenarios is shown in Figure 4. The Wald *F* test with the Satterthwaite approximation can keep the type I error rates to nominal level as long as the number of clusters is greater than 30. The method tends to be conservative when the cluster number is lower than 30, and the conservativeness becomes more severe with the increase of the cluster size variation. As shown in Figure 4, the Wald *F* test with the Satterthwaite approximation only keeps the observed type I error rates close to nominal level under the balanced design (*cv* = 0) when the number of clusters is smaller than 20. As cluster size variation increases, the observed type I error rates drop dramatically. The conservative type I error rates caused by the increased variation of cluster size can be diminished by increasing the total number of clusters, but not to nominal level. The conservativeness definitely will preserve the validity of the Wald *F* test, but it may decrease the statistical power of the test.

The K-R method inflates the marginal variance-covariance matrix and then applies the Satterthwaite method for the DDF approximation. Because we only test the null hypothesis of intervention effect, the K-R method has the exactly same DDF approximation as the Satterthwaite method. Its small sample performance with regarding the type I error rate under various CRT scenarios is very similar to the Satterthwaite method, but a little more conservative due to the standard error inflation, as shown in Figure 5. Therefore, this method will preserve the validity of the Wald *F* test; however, its conservativeness may cause power loss, especially when considerable cluster size variation.

In summary, the number of clusters and the cluster size variation, rather than ICC and the average cluster size, play important roles on the type I error control for the five DDF approximation methods in GLMM analysis to test the null intervention effect under the framework of CRTs with binary outcomes. When the cluster number is smaller than 30, neither Residual nor Containment method should be used due to the inflated type I errors. In contrast, both Satterthwaite and K-R methods tend to be conservative, especially when a considerable cluster size variation exists. Our simulations suggest that the B-W method preserves the type I error rates to nominal level in the GLMM analysis of CRTs with a small number of few clusters and is robust cluster size variation. It should be noted that only binary outcomes are studied here and the aforementioned results may not be directly applicable to outcomes with different distributions.

### Statistical power of Wald *F* tests

**Figure 6:**
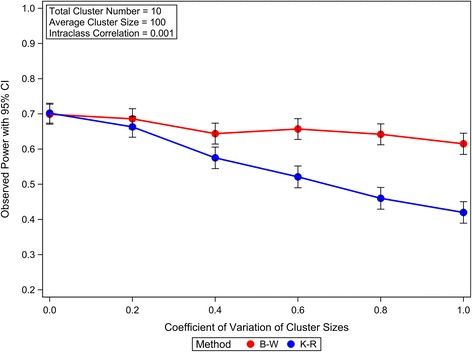
The effects of variation of cluster sizes on the power of GLMM Wald *F* tests in analyzing CRTs with few heterogeneous clusters. The observed powers and the 95% confidence intervals are calculated from 1000 independent simulation replicates.

**Figure 7:**
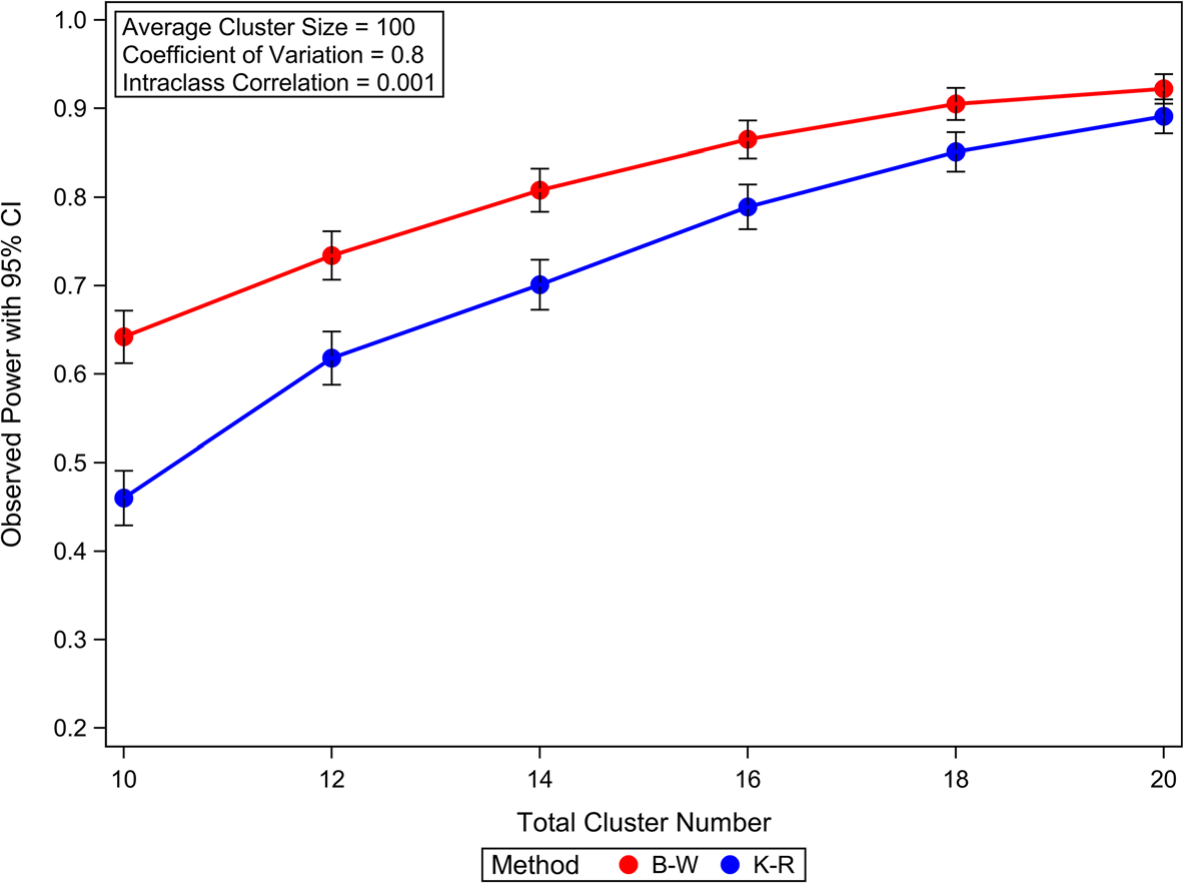
The diminished power loss of GLMM Wald *F* test with Kenward-Roger approximation of denominator degrees of freedom with the increase of cluster number in analyzing CRTs with few heterogeneous clusters. The observed powers and the 95% confidence intervals are calculated from 1000 independent simulation replicates.

To illustrate how the cluster size variation affects the statistical power of GLMM analysis of CRTs with few heterogeneous clusters, the empirical powers of Satterthwaite method, K-R method and B-W method are calculated and compared under different CRT scenarios. Our simulations suggest that the empirical powers of Satterthwaite method are very close to those of K-R method; however, both of these two methods are less powerful than the B-W method. The power comparison is illustrated in the Figure 6, in which the empirical powers for K-R and B-W methods are plotted under different variation of cluster size in analyzing the simulated CRTs assuming: 1) 10 total clusters equally allocated in two arms; 2) average cluster size of 100; 3) the intraclass correlation equal to 0.001; 4) proportion of events in control arm is 0.25; and 5) the odds ratio equal to 1.5. Similar patterns of the empirical powers for these methods are also observed under different CRT settings (data not shown). Because the empirical powers of Satterthwaite method are very close to those of K-R method, only the empirical powers of K-R method are plotted for the illustration. Although the empirical powers decrease with the increase of the cluster size variation for all the three methods, Satterthwaite and K-R methods are more sensitive to the cluster size variation as illustrated in Figure 6. The greater power loss of Satterthwaite and K-R methods could be partially explained by their increased conservativeness with the increased variation of cluster sizes, as shown previously. The power difference between B-W method and K-R method (or Satterthwaite method) can be diminished by increasing the cluster number, as shown in the Figure 7. This phenomenon is consistent with the observation that the conservativeness of Satterthwaite and K-R methods caused by the variation of cluster sizes can be diminished by increasing the cluster number.

In summary, the B-W method is statistically more powerful than the Satterthwaite or K-R method in analysing CRTs with heterogeneous clusters, especially when the cluster number is small and the variation of cluster size is large.

### Real data illustration

The GLMM is used for the data analysis and the small sample inferences of intervention effects with different DDF approximations are listed in Table 2. The estimated ICC in this study is 0.026. The Residual and Containment methods specify large DDFs and consequently generate the smallest p values (p = 0.034 for both methods). In contrast, the Satterthwaite and K-R methods give the smallest DDF estimation and the most conservative p values (0.046 and 0.047, respectively). Although the Satterthwaite and K-R methods have the same approximations of DDF in this analysis, the K-R method has the smaller *F* value and more conservative p value due to its further inflation of the standard error estimation (0.4507 vs 0.4485). Based on our simulation results and the facts of large variation of cluster sizes and small number of clusters in this trial, we may expect that the Residual and Containment approximations will generate liberal tests while the Satterthwaite and K-R approximations will generate conservative tests in contrast. However, the B-W DDF approximation will give the best small sample inference of intervention effect. We may conclude that the women in intervention practices were estimated to be about 2.6 times more likely than those in the control practices to attend for subsequent breast screening.

**Table 2:** **GLMM small sample inferences of intervention effects on women’s attendance at breast screening with different denominator degrees of freedom approximations**

**Method**	**Intervention estimate**	**Standard error**	***F*** **value**	**Numerator DF**	**Denominator DF**	***P*** **value**
Residual	0.9517	0.4485	4.50	1	2062	0.0340
Containment	0.9517	0.4485	4.50	1	2038	0.0340
B-W	0.9517	0.4485	4.50	1	24	0.0444
Satterthwaite	0.9517	0.4485	4.50	1	20.85	0.0460
K-R	0.9517	0.4507	4.46	1	20.85	0.0469

## Discussion

When the GLMM is used in the analyses of CRTs, the null hypothesis of the treatment effect can be tested using the Wald statistics by dividing treatment mean squares by the appropriate error mean square to form a variance ratio with an *F* distribution. The numerator degrees of freedom can be specified by the number of fixed effect contrasts being considered, but the determination of suitable DDF must be estimated in the unbalanced mixed models [24]. In this study, we compare and contrast the small sample performances of five methods of DDF approximation for the GLMM Wald *F* test under the framework of CRTs regarding the type I error and power. Our simulation results suggest that the B-W method maintains the type I error rates to the nominal level even when the number of clusters is as low as 10, and is robust to the variation of the cluster sizes. The Residual and Containment methods inflate the type I error rates when the cluster number is small (<30) and the inflation becomes more severe as the variation of cluster sizes increases. In contrast, the Satterthwaite and K-R methods may provide tests that are too conservative when the cluster number is small (<30) and the conservativeness becomes more severe with the increase of cluster size variation. However, the inflation or deflation of the type I error rates caused by the imbalance of the cluster sizes can be diminished by increasing the number of clusters. When the cluster number is greater than 30, all the methods are robust to the variations of the cluster sizes.

The Between-Within method is proposed for the small sample adjustment to the longitudinal repeated measures [18]. This method divides the residual degrees of freedom into between-cluster and within-cluster values and assigns a between-cluster denominator degrees of freedom to a the fixed effect that does not change within clusters. In the GLMM analyses of CRTs, the intervention effect does not change within clusters, and then a between-cluster denominator degrees of freedom, *K* − 2, is assigned to the Wald *F* test of the null hypothesis of intervention effects. This method is proposed for the longitudinal repeated measures and is supposed to be valid only for the balanced design; however, in our simulations, this method preserves the type I error rates to a nominal level and it is robust to the small number of clusters and the variation of cluster sizes.

The Residual method does not take the correlation into account and is only valid for the independent outcomes. It is not surprising that the Wald *F* test with the Residual approximation of DDF has the inflated type I error rates in the GLMM analyses of CRTs. The Containment method mimics the classical degrees of freedom rules for balanced ANOVA situations, and is the default method for the SAS procedures PROC MIXED and PROC GLIMMIX when the random statements are specified [11]. In the analyses of CRTs, the intervention effect is usually considered as the fixed effect so that the DDF of the single parameter Wald *F* statistic for the intervention effect by the Containment method will be approximated in the similar way as the Residual method. Our simulation results show that, like the Residual method, the Containment method inflates the type I error rates to test the null hypothesis of intervention effect in the GLMM analyses of CRTs. Therefore, neither of these two methods should be considered in the GLMM analyses of CRTs.

Both Satterthwaite and K-R methods estimate the DDF from the data through matching the first two moments of the Wald *F* statistics and the approximating *F* distribution [13,14]. Compared with the Satterthwaite method, the K-R method further adjusts the covariance matrix for the fixed effects parameters that accommodates the uncertainty in the covariance matrix [14]. Since their appearance, these two methods, especially the K-R method, have been favored by many studies under the random complete block design, split plot design and repeated measures design [12,15]. Spilke et al. [12] conclude that the Satterthwaite method provides good type I error control and the K-R method gives the best type I error control by reducing the bias of the estimated variance-covariance matrix of fixed effects parameters under random complete block design. Schaalje et al. [15] investigate the repeated measures design and conclude that the K-R method works as well as or better than the Satterthwaite method in maintaining the type I error rates close to the nominal level. In contrast to these previous studies, our simulation results suggest that both Satterthwaite and K-R methods tend to be overly conservative, especially when a considerable variation of cluster sizes exists, under the framework of CRTs and a binary outcome. Not surprisingly, the conservativeness causes greater power loss in analyzing the CRTs with few heterogeneous clusters. Unfortunately, large variation of cluster sizes is common in CRT design and the so caused power loss could be very costly if the Satterthwaite or K-R method is going to be used in the analysis.

The variance-covariance structures have been shown in many studies to affect the small sample performances of different denominator degrees of freedom approximations [12,15,18]. Under the CRT framework, the compound symmetry is the most commonly accepted variance-covariance structure and therefore the only consideration in our study. In actual practice, the intraclass correlation among the same cluster is low and usually less than 0.05 [4]. Under the range (0.001, 0.01, 0.05 and 0.1) investigated in this study, we find that the intraclass correlation has little effect on the small sample performances of all the five methods we evaluated. However, for those CRTs with a more complicated correlation structure, such as the CRTs with binary longitudinal outcomes, the small sample performances of the DDF approximations need further evaluation. Another limitation of this study is that only binary outcomes are considered and the small sample performances of the five DDF methods on other types of outcomes (count, time-to-event, etc.) need further investigations.

## Conclusion

In conclusion, we compare the small sample performances of five DDF approximation methods in GLMM to test the null hypothesis of intervention effect under the framework of CRTs with binary outcomes, and find that the B-W method outperforms the other four methods by its ability to preserve the type I error rates to nominal level and its relatively higher statistical power. Therefore, the B-W method should be recommended in the GLMM analyses of CRTs with few heterogeneous clusters.
